# Metaphase FISH on a Chip: Miniaturized Microfluidic Device for Fluorescence *in situ* Hybridization

**DOI:** 10.3390/s101109831

**Published:** 2010-11-02

**Authors:** Indumathi Vedarethinam, Pranjul Shah, Maria Dimaki, Zeynep Tumer, Niels Tommerup, Winnie E. Svendsen

**Affiliations:** 1 DTU-Nanotech, Department of Micro and Nanotechnology, Technical University of Denmark, Kgs. Lyngby 2800, Denmark; E-Mails: Maria.Dimaki@nanotech.dtu.dk (M.D.); Winnie.Svendsen@nanotech.dtu.dk (W.E.S.); 2 Wilhelm Johannsen Centre for Functional Genome Research, Department of Cellular and Molecular Medicine, The Panum Institute, University of Copenhagen, Blegdamsvej 3, 2200 Copenhagen, Denmark; E-Mails: zet@kennedy.dk (Z.T.); ntommerup@sund.ku.dk (N.T.); 3 Kennedy Center, Gl. Landevej, 2600 Glostrup, Denmark

**Keywords:** Fluorescence *in situ* Hybridization (FISH), lab on chip, genetic analysis, cytogenetics, chromosome spreading, metaphase FISH, chromosomal translocations

## Abstract

Fluorescence *in situ* Hybridization (FISH) is a major cytogenetic technique for clinical genetic diagnosis of both inherited and acquired chromosomal abnormalities. Although FISH techniques have evolved and are often used together with other cytogenetic methods like CGH, PRINS and PNA-FISH, the process continues to be a manual, labour intensive, expensive and time consuming technique, often taking over 3 5 days, even in dedicated labs. We have developed a novel microFISH device to perform metaphase FISH on a chip which overcomes many shortcomings of the current laboratory protocols. This work also introduces a novel splashing device for preparing metaphase spreads on a microscope glass slide, followed by a rapid adhesive tape-based bonding protocol leading to rapid fabrication of the microFISH device. The microFISH device allows for an optimized metaphase FISH protocol on a chip with over a 20-fold reduction in the reagent volume. This is the first demonstration of metaphase FISH on a microfluidic device and offers a possibility of automation and significant cost reduction of many routine diagnostic tests of genetic anomalies.

## 1. Introduction

During the last decade microfluidic techniques have evolved into a new genre of research areas targeting integration of laboratory protocols into miniaturized devices called lab on chip (LOC) or micro total analysis systems (μtas) [1–5]. The ability to control small sample volumes on micro-sized devices is immensely appealing for techniques involving handling of ultra small volumes of cells or other analytical samples [6]. This has spurred an exponential growth in the number of research articles published in the field of LOC systems targeting complex biological protocols to benefit from the low volume, high throughput and low cost features provided by the microfluidic devices [6–9]. We have created a novel metaphase FISH chip to benefit from the low dead volumes associated with microfluidic devices as FISH protocol reagents and commercial probes are more expensive than gold (over $400 for 100 μL of probe used in this work) [10,11].

FISH is a sensitive diagnostic cytogenetic tool routinely used to visualize numerical and structural chromosomal aberrations [12–20]. The traditional FISH protocol includes steps like immobilization of interphase nuclei or metaphase chromosomes, probe labeling, RNAse treatment, denaturation of chromosomal and probe DNA, hybridization, post hybridization wash and image processing. FISH is routinely used by cytogeneticists in applications ranging from chromosome labeling and mapping, identification of gene expression sites, tissue analysis, mRNA synthesis tracking, tumour genetic alterations monitoring, identifying infections from viruses and other diagnostic pathological applications including cancer e.g., leukemia [21,22].

Interphase and Metaphase FISH are two types of commonly used chromosomal FISH techniques. Each has their specific applications and advantages. Interphase FISH is used to identify numerical abnormalities as well as specific structural abnormalities. Thus, Interphase FISH depends on a specific probe, e.g., to detect known translocations, microdeletions or specific chromosomes and hence can only be used to address questions for which DNA probes are available. Lack of conformity of interphase FISH is a major disadvantage when using this technique for prenatal diagnostics [23]. On the other hand, metaphase FISH can be used to visualize the insertion, deletion or other rearrangement involving a specific region of the genome, with a resolution determined by the probe used [17]. Metaphase FISH can be performed on samples with unknown translocations by targeting all the chromosomes using multi-color FISH probes [24], derived from plasmids, cosmids, bacterial artificial chromosomes (BAC) and yeast artificial chromosomes (YAC) [18]. Recently Interphase FISH was demonstrated on a microfluidic device which highlights the obvious benefits of miniaturizing and automating the FISH protocol in a microfluidic system [10,11,25]. But metaphase FISH protocol has been elusive owing to the difficulty of handling chromosomes on a chip and fixing the chromosomes on a closed microfluidic device [25]. As a result, in spite of FISH being a very powerful cytogenetic tool, it continues to be an expensive and reasonably time consuming method. There is clearly a need for replacing the traditional method with a fast and low-cost method making this technique widely available and easy to handle.

Our efforts have been directed at designing a miniaturized protocol for performing metaphase FISH in a controlled manner on a microfluidic device. It is widely known that the results of classical banding techniques, FISH analysis or Comparative Genomic Hybridization (CGH) are dependent on the quality of the metaphase spreads [26]. Hence the result of any FISH analysis depends on consistency of spreading of chromosomes [27]. During the 90s metaphase spreading techniques were widely investigated and many labs developed their own version of optimized standard methods [28–34]. This led to a number of theories on what determines the quality of metaphase spreads on the glass slide, including the distance and the angle of dropping of the fixed cells onto the slide, the diameter of the pipette, the evaporation of the fixative, the temperature of the slide, the hypotonic treatment of the chromosomes, and the whole air drying process [27,35,36]. Renewed interest has seen a number of attempts towards making devices for preparation of chromosome spreads, which rely on controlled angle and conveyors [37], temperature gradient and humidity [26] and most recently the chromosome dropper tool which relies on dropping angle and height for fixing metaphase spreads on glass slides [38]. But none of these three devices are remotely close to the miniaturized size we are targeting or give any new insights into the mechanism of spreading compared to what was already published in the 90s. Through literature review and our own preliminary experiments we found that the basis of chromosome spreading is rooted in the optimum rate of evaporation of the fixative from the glass slide. Hliscs *et al.* suggests that the mechanism of chromosome spreading is a slow process, which leads to stretching of chromosomes via flattening [39]. Spurbeck *et al.* mentioned that in the process of spreading the fixative evaporates, leading to build up of surface tension causing the metaphase cell to flatten, which eventually leads to bursting of the cell membrane and spreading of the chromosomes [27]. Henegariu *et al.* also concluded that dropping of chromosomes from a height doesn’t improve the spreading [26]. Hence, we concluded that in order to realize a micro-splashing device which produces reliable metaphase spreads on a glass slide sufficient for conducting routine FISH analysis; we need to incorporate a mechanism to allow for optimum evaporation of the fixative leading to stretching of the chromosomes and flattening of the cells. This could be aided by environmental factors like temperature of the slide and humidity but the focus has to be on maintaining an optimum rate of fixative evaporation.

In order to account for that, we devised a novel splashing device with open chamber, which allows for easy evaporation of the fixative. The device provides 11 mm dropping height with two inlets—one for cold water and one for the fixed mitotic cells suspension. Apart from the splashing device, a novel metaphase FISH protocol was developed using a microFISH device. This microFISH device provides the possibility of replacing the traditional Coupling Jar and turning it into a miniaturized microfluidic device. We have looked into possible replacements of glass slides with various common polymers but found them unfavorable for FISH protocol on mainly three counts. Firstly, the spreading of the metaphase chromosomes on the glass slides is highly aided by the surface properties of the glass like contact angle and free surface energy. Secondly, polymers exhibit auto-fluorescent behaviour which hinders the analysis due to interference with the FISH probe signals. Finally, some polymers like PMMA couldn’t withstand the fixative used for fixing the metaphase chromosomes and cracked on contact. Hence, we found it ideal to continue using the traditional microscope glass slides as substrate which also offers the benefits of easy integration into existing workplace protocols in cytogenetic labs. In order to minimize the time of fabrication of the microFISH device, an easy protocol for rapid bonding based assembly has been developed. With this the glass slide can be turned into a microFISH device in a matter of seconds by using an adhesive tape-based stencil and bonding technique (described later in Fabrication section). The ease of operation and handling in this protocol has been targeted to allow non-technical personnel to easily conduct these tests. The rapid fabrication protocol leaves room for conducting multiple tests simultaneously. In addition, the novel protocol allows for an over 20-fold reduction in reagent volume, which is significant considering the costs of commercially available probes. We hope that this first demonstration of metaphase FISH on a chip, will spur renewed efforts in automating metaphase FISH on a chip leading to wider access to low cost, reliable and fast genetic diagnostics in clinical environments. Although the presented device has been developed for metaphase FISH, we feel that the protocol could also be applied for Interphase FISH.

## 2. Experimental Section

### 2.1. Materials and Chemicals

Polymethylmethacrylate (PMMA) sheets procured from Nordplast (Denmark) were used to fabricate the Splashing device; Polydimethylsiloxane (commonly known as PDMS, Sylgard 184, Dow Corning, USA) was used to fabricate the microFISH device. Glass slides (SuperFrost), syringes and silicone tubing were obtained from traditional suppliers (Sigma-Aldrich, VWR). SU8-2075 resist was ordered from Microchem (Germany). Double-sided adhesive tape AR100 (50 μm thickness) was procured from Adhesive Research Inc. (Ireland). All the reagents used in the FISH experiment were purchased from Invitrogen (Paisley, UK). 1 μL of RNAse (10 μg/μL) stock solution was mixed with 99 μL of 2 × SSC for removal of RNA. The probe was purchased from Kreatech Diagnostics (Amsterdam, The Netherlands) and it was used as recommended by the manufacturer. 20 × SSC (sodium citrate, sodium chloride) was purchased from G-Biosciences (USA) and further dilutions were made using Milli-Q water. The stock of absolute ethanol was used to make dilutions (50%, 60%, 90%, 99% ethanol). 4′,6-diamidino-2-phenylindole (DAPI, Invitrogen) was used as a counterstain used for colouring the chromosomes. Blood sample of a female patient was received from the Wilhelm Johannsen Centre for Functional Genome Research, Department of Cellular and Molecular Medicine. Cold water used for spreading chromosomes was kept at 4 °C.

### 2.2. Apparatus

A micromilling machine (Folken Industries, Glendale, USA) was used for milling the splashing device, 50 W CO_2_ Laser Machine (Synrad Inc., USA) was used for ablating the adhesive tapes, a photolithographic spinner and aligner from Carl-Zeiss were used for fabricating the master mould for PDMS microFISH device and a Zeiss Axio Observer Z1 Fluorescent microscope was used for analysis of the spreads and FISH signals.

### 2.3. Fabrication

**Figure 1.**
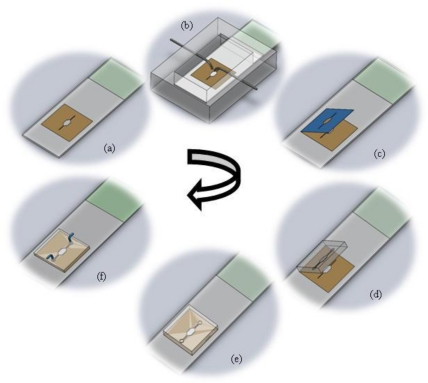
Schematic Protocol for splashing the metaphase spreads followed by rapid assembly of the microFISH device. **(a)** Bonding of the double-sided adhesive tape stencil on the glass slide; **(b)** Spreading of the metaphase spreads in the splashing device; **(c)** Peeling off the top cover of the double-sided tape stencil to leave only the spreads in the centre of the glass slide and to expose the adhesive layer to bond the PDMS microFISH lid; **(d)** Aligning the PDMS lid on to the adhesive tape; **(e)** Assembly of the microFISH by bonding the PDMS lid on to the tape using gentle pressure; **(f)** Making the interconnection holes and connecting the syringes for world-to-microFISH device fluidic connections.

The fabrication protocol has been summarized in Figure 1. The figure details the protocol for splashing metaphase spreads on the glass slide using the splashing device followed by the rapid fabrication of the microFISH device to perform the metaphase FISH analysis protocol. The details of the fabrication protocol will be explained in the following subsections.

#### 2.3.1. Glass Slide with Stencil

**Figure 2.**
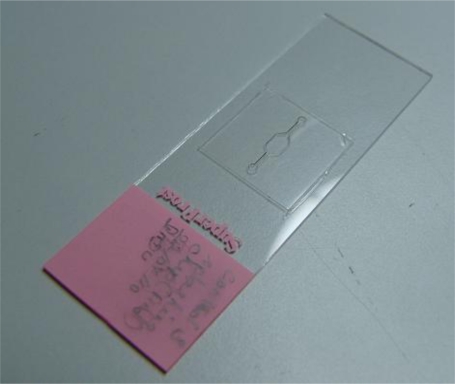
Glass Slide with Laser Ablated Tape Stencil.

The stencil for localization of metaphase spreads is created using a double-sided medical grade tape. The double-sided adhesive tape is fabricated using a laser ablation process with a CO_2_ laser [40–42]. The laser is operated at 20 W power at 250 mm/s laser velocity using resolution of 800. The operating parameters are set using the Winmark software (Synrad Inc, USA). The design of the tape corresponds to the design of the microFISH device (Figure 2). When the tape is ablated using the CO_2_ laser, one side of the tape cover is peeled and the tape is bonded to the centre of the glass slide as shown in Figure 2. This tape-glass slide complex acts as a stencil when the glass slide is used in the splashing chip to create metaphase spreads. The localization of the metaphase spreads is achieved by removing the top cover of the tape which allows selective patterning on the glass slide in the centre where the tape is fully ablated using the CO_2_ laser for creating the microFISH chamber.

#### 2.3.2. Splashing Device

**Figure 3.**
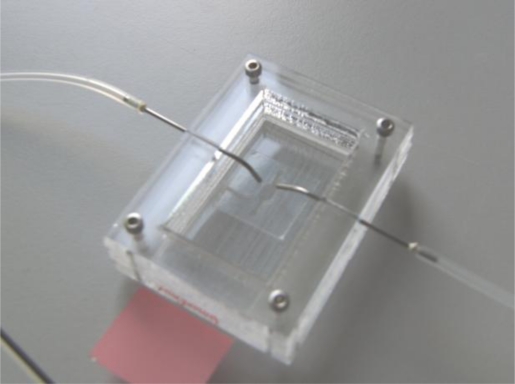
The splashing device fabricated with two layers of PMMA. The glass slide with tape stencil is inserted into the sliding chamber in the lower PMMA and the top PMMA plate has a centre open chamber for fixative evaporation and two inlets for glacial water and fixed mitotic cell suspension.

The splashing device is fabricated in two PMMA layers by a micro-milling process. The bottom part contains the sliding chamber for glass slide insertion and the top lid contains the open chamber for evaporation of the fixative. It also contains the two inlet ports for fixed chromosomes and cold water (Figure 3). The syringes are slightly bent towards the end to focus the water and chromosome suspension on to the centre of the stencil which exposes the glass slide. The two PMMA layers are either bonded together using thermal bonding [42] or screwed together as shown in Figure 3.

#### 2.3.3. Master for PDMS Chip and Moulding of the PDMS MicroFISH Chip

**Figure 4.**
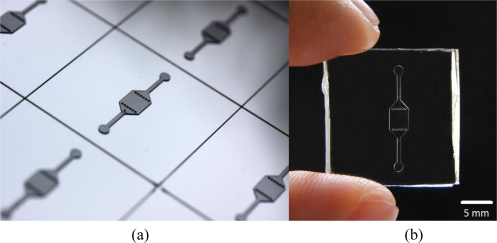
**(a)** Master for moulding PDMS microFISH device lid fabricated in SU-8 photoresist over silicon wafer; **(b)** PDMS microFISH device lid moulded using PDMS elastomer.

The master for the microFISH device lid was prepared in a cleanroom using a traditional photolithography process. The structures were created on a Silicon wafer in SU-8 photoresist using negative patterning. The recipe for creating the master is shown in Table 1. This master can be used several times for moulding the microFISH device lid (Figure 4). The microFISH device lid is moulded in PDMS using traditional soft lithography techniques described in [43] and peeled away from the Silicon/SU-8 master. The final PDMS microFISH device lid is shown in Figure 4. The lid is then bonded on the glass slide with metaphase spreads for assembling the microFISH device.

#### 2.3.4. Assembly of the MicroFISH Device

**Figure 5.**
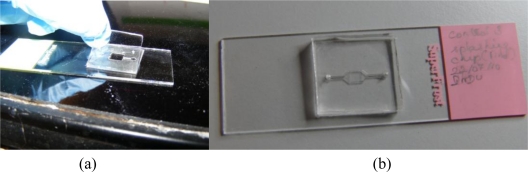
**(a)** Peeling off the top cover of the double-sided tape stencil to expose the silicone adhesive layer; **(b)** Assembled microFISH device by bonding the microFISH device lid on to the silicone adhesive layer.

The double-sided adhesive tape stencil used for spreading chromosomes is now used as a bonding layer for assembling the microFISH device. Peeling the top cover off the tape provides a silicone bonding layer for the microFISH device lid (Figure 5). The PDMS lid is irreversibly attached to the glass slide using the silicone adhesive layer by gently applying pressure across the PDMS lid (Figure 5). Once the device is assembled the holes are made in the PDMS lid for access ports to create interconnections.

#### 2.3.5. Interconnects

**Figure 6.**
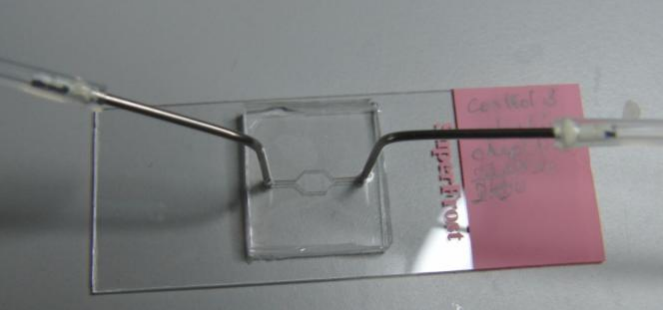
Fully assembled microFISH device with interconnects and tubings to connect it to the syringe pump.

The access holes in the microFISH device lid are made on the top side in the PDMS lid. The holes are made using a 22 gauge syringe needle (0.7 mm outer diameter). The interconnects are formed by inserting an 18 gauge syringe (1.2 mm outer diameter) with a bigger outer diameter than the interconnection holes, which forms a tight seal into the microFISH device. The syringes are connected to silicone tubing with inner diameter 1 mm and outer diameter 3 mm to provide the world-to-microFISH device contacts (Figure 6). In order to test the device, the silicone tubings are connected with syringe pumps (not shown in the figure) which provides the pressure needed to actuate the reagents and probes through the microFISH device.

### 2.4. Procedures

#### 2.4.1. Splashing Protocol

**Figure 7.**
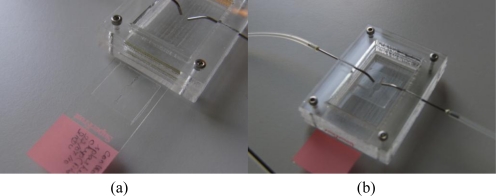
**(a)** Sliding of the glass slide with stencil into the splashing device; **(b)** Splashing device with glass slide. The stencil is positioned under the syringes for splashing glacial water followed by fixed mitotic cells for spreading.

The chromosome suspensions used for creating the metaphase spreads were prepared by standard methods [22]. Peripheral blood lymphocyte cultures were prepared and treated with 75 mM KCl hypotonic solution for 5 minutes during the harvesting stage. Claussen *et al.* signified the importance of proper hypotonic treatment for achieving good quality metaphase spreads [31]. The hypotonic treatment causes the mitotic cells to swell leading to the chromosomes moving to more peripheral locations, which allows for evenly spreading of the chromosomes during fixative evaporation. After the mitotic cells are treated with the hypotonic solution they are fixed using the fixative (methanol/acetic acid, 3:1). The fixative is added drop by drop to the cells all the while mixing thoroughly on a vortex. To compare the spreads achieved using the traditional dropping method and the splashing device, control slides were prepared using the manual dropping method and test slides were prepared using the splashing device. Before dropping the cell suspension on the control slides, the slides are kept in distilled water at 4 °C. On the other hand, the test slides were prepared using the splashing device, where one drop of glacial water was dropped on to the slide followed by a drop of the fixed mitotic cell suspension through the two dedicated inlets (Figure 7). The slides were allowed to air dry in the open chamber of the splashing device. As a final step, both test and control slides were heated at 75 °C degrees for 3 min, which improves the fixation of the chromosomes to the slide.

#### 2.4.2. FISH Protocol

The FISH protocol was conducted on both the control and test slides using the microFISH device. After assembling the microFISH device using the glass slides with chromosome spreads, the optimized FISH protocol was conducted on all the slides. The inlet of the microFISH device was connected with a syringe pump for treating the metaphase spreads with the FISH reagents and probes. The specific temperatures associated with the FISH protocol were provided by a hot plate setup. Firstly, RNAse (10 μg/μL) was delivered through the inlet. Following the RNAse treatment, the microFISH device was kept in the humidity chamber at 37 °C for one hour. Later 2 × SSC was loaded at room temperature for washing the metaphase spreads. The microFISH device was then dehydrated with increasing percentages of ethanol (70%, 80% and 99%), and the device was heated at 75 °C to denature the chromosomal DNA for 5 minutes. Simultaneously, probe DNA was denatured at 75 °C for 5 minutes in the water bath. Then the probe was entered into the device and the inlet and outlet were closed to avoid evaporation of the probe. The microFISH device was incubated in the humidity chamber at 37 °C overnight for hybridization. Subsequently, 50% formamide was delivered as post hybridization wash at 42 °C. Final washing was performed with 0.1 × SSC at 60 °C, 4 × SSC and 1 × PBS at room temperature. The details of the conventional FISH protocol and optimized FISH protocol can be found in Table 2.

#### 2.4.3. Analysis

DAPI was applied to the hybridized targets on the test slides in microFISH chamber and in case of control samples, slides were mounted with cover slips. The Zeiss AxioObserver Z1 fluorescent microscope was used to view and analyze the samples for counting the metaphase spreads and the FISH hybridization signals. The chromosomes spreads on the control and test slides were counted manually using the traditional method.

## 3. Results and Discussion

### 3.1. Splashing Device and Metaphase Spreads

**Figure 8.**
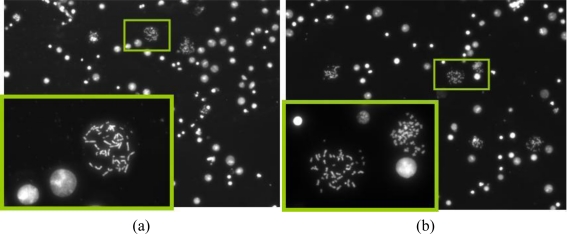
**(a)** Metaphase spreads stained with DAPI on the control slides prepared using the traditional dropping method (Inset—40× resolution); **(b)** Metaphase spreads stained with DAPI on the test slides prepared using splashing protocol (Inset—40× resolution).

**Figure 9.**
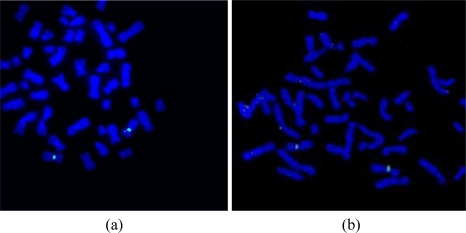
**(a)** FISH analysis on the control slide. The two green dots highlight the two X chromosomes in the female sample; **(b)** FISH analysis on the test slide. The two green dots highlight the two X chromosomes in the female sample.

The motivation behind the splashing device was to create a microfluidic device which can provide reliable and sufficient number of metaphase spreads on a glass slide. After spreading the chromosomes on the slide using the traditional method and the splashing device, the slides were stained with DAPI. The metaphase spreads were counted manually in the fluorescence microscope at 20× magnification. Figures 8 and 9 show the images of the metaphase spreads obtained using the conventional method and splashing device respectively. In order to validate the splashing device protocol, we conducted tests using two different cell suspension samples. Table 3 shows the comparison of average spreads obtained using the two techniques.

Even though the average numbers of spreads obtained by using the splashing device are comparatively lower, we could obtain sufficiently good chromosome spreads in order to perform the FISH analysis. The lower chromosome spread counts may be due to the splashing conditions such as height, temperature and humidity as described in the introduction. While in average the number of chromosome spreads was significantly lower in the splashing device, it must be noted that in certain samples, we found the number of chromosome spreads to be higher compared to the control slides. Considering that the splashing experiments were not studied in detail, we believe that the splashing protocol can be optimized by altering and controlling the various other factors like temperature, humidity, *etc*.

Moreover, the simple fabrication protocol of the splashing device using micro milling of PMMA sheets allows us to control the height of splashing if needed by addition of more PMMA layers to increase the height of the splashing syringe. The integrated stencil and bonding layer of the adhesive tape allows for localization of the metaphase spreads on the glass slide in the splashing device. Thus, the splashing device not only provides an ability to reliably create metaphase spreads on glass slide but also provides a possibility of automating the image analysis procedure in the future due to ease of finding the metaphase spread using software and an automated stage. A number of companies (Zeiss, BioView and Vysis) and research groups are working on optimizing the data acquisition protocols as this continues to be a major issue after the FISH protocol [44,45]. Automation of the analysis procedure is currently an on-going work where we are aiming to divide the microFISH device chamber into smaller segments to create smaller FISH microchambers corresponding to the microscope objective.

### 3.2. FISH Protocol Results

After the preparation of the chromosome spreads, FISH analysis was performed on the peripheral blood lymphocyte chromosomes using an X chromosome centromeric probe. A female patient sample was used in order to show the validation of the microFISH device protocol and the results obtained with XX chromosomes (Figure 9(b)) confirm the successful FISH analysis result. The conventional FISH protocol was also performed on the slides to compare and evaluate the microFISH device efficiency (Figure 9(a)). As can be seen in Table 2, the reagent volume needed in traditional FISH analysis is 327 mL, but now has been reduced to only 14 mL, which is a major step in reducing the costs associated with FISH analysis. As mentioned earlier, the most expensive reagent in traditional FISH analysis is the probe (costing approx. $90 per test). In the microFISH protocol the volume of this has been reduced to half (highlighted in yellow in the Table 2). This alone cuts the cost associated with conducting the routine FISH analysis in half. Considering the cost of fabricating these microFISH devices is less than $2, these successful FISH results will give a major push towards making genetic analysis a routine screening test.

## 4. Conclusions

We have successfully demonstrated the first metaphase FISH on a microfluidic device. This work also presents an alternative method for slide preparation and hybridization of FISH probes. The achievements gained in the present study will be used in further improvement of the methodology and aim to develop a completely automated system for performing miniaturized FISH on chip. The micro splashing device designed for spreading metaphase chromosomes on a glass slide provides more reliable and easy alternative for creation of metaphase spreads. The rapid and easy assembly protocol for the microFISH device allows for quick transformation of a simple glass slide into a microFISH device, which makes it an ideal solution for integration into existing work routines at cytogenetic labs. Our current efforts are focused on further miniaturization of this device, which will offer significant benefits with respect to sample preparation and reduction in reagent costs. We are also working towards improving the chromosome spreading protocol by further miniaturization of the splashing chamber and localized micro-spotting of fixed cells, optimizing the hybridization efficiency using temperature and electro-kinetic effects, automating the protocol by incorporation of reagent reservoirs and improving the analysis by developing automated software for data acquisition and metaphase spread recognition. In the longer run, we envisage to create a chromosome total analysis system providing a more efficient and cheaper solution to the traditional protocol and enable faster diagnosis.

## Figures and Tables

**Table 1.** Recipe for fabrication of SU-8 mould using negative photolithography process.

**Step**	**Type**	**Parameters**

1	Spin-coating of SU-8	Acceleration: 200 rpm/s, Speed: 1,000 rpm, Time 40 seconds
2	Soft Bake	Temperature: 50 °C, Time: 5 hrs, Ramp up Time: 15 mins
3	Exposure	Time: 30 seconds Type: Soft
4	Post Exposure Bake	Temperature: 50 °C, Time: 5 hrs, Ramp up Time: 15 mins
5	Development	First: 4 minute, Final: 1 minute

**Table 2.** Comparison of conventional FISH method and microFISH protocol showing over 23 fold reduction in total reagents used and a 2 fold reduction in the probe volume (highlighted in yellow) which is the most expensive reagent for conducting FISH analysis. All steps of the FISH protocol are presented and in case of the microFISH protocol the flow rates used for the FISH reagents are also presented.

		**Conventional protocol**	**microFISH protocol**	

**Step No.:**	**Name of the step**	**Vol**	**Vol**	**Flow rate**

1	RNAse	100 μL	25 μL	5 μL/min
2	Wait 60 mins			No flow
3	Wash 2 × SSC	75 mL	3 mL	200 μL/min
4	70% Alcohol	25 mL	1 mL	200 μL/min
5	90% Alcohol	1 mL	1 mL	200 μL/min
6	100% Alcohol	1 mL	1 mL	200 μL/min
7	Drying-Air			NA
8	70% formamide	25 mL		NA
9	90% Alcohol	25 mL		NA
10	100% Alcohol	25 mL		NA
11	Probe	10 μL	5 μL	2 μL/min
12	Hybridisation time	Overnight	Overnight	No flow
13	50% Formamide	75 mL	3 mL	200 μL/min
14	0.1 × SSC	25 mL	3 mL	200 μL/min
15	4 × SSC	25 mL	1 mL	200 μL/min
16	1 × PBS	25 mL	1 mL	100 μL/min
17	DAPI	15 μL	5 μL	2 μL/min

**Total Reagent volume**		**327.2 mL**	**14.1 mL**	

**Table 3.** Average number of metaphase spreads found in the microFISH chamber (3 × 3 mm^2^) using manual dripping method and our splashing device protocol.

	**Sample 1**	**Sample 2**
**Control Slide**	**41**	**52**
**Splashing device**	**29**	**34**
